# Modulation of the growth performance, amino acid digestibility, and jejunal integrity of broiler chickens by dietary inclusion of raw potato starch or high-amylose corn starch in a mixed-*Eimeria* challenge model

**DOI:** 10.1016/j.psj.2025.105963

**Published:** 2025-10-10

**Authors:** Iyabo W. Oluseyifunmi, Oluyinka A. Olukosi

**Affiliations:** Department of Poultry Science, University of Georgia, Athens, GA 30602, USA

**Keywords:** Raw potato starch, High-amylose corn starch, Eimeria, Broiler chickens

## Abstract

A mixed *Eimeria* spp. challenge model was used to evaluate the potential ameliorative effects of dietary resistant starches (**RS**) on growth performance, ileal amino acid digestibility (**AID**), and gut integrity in broiler chickens during *Eimeria* challenge. A total of 1,056 day-old male broiler chicks were assigned to 8 treatments in a 4 × 2 factorial arrangement. Diets included 0 RS (control), 25, or 50 g/kg raw potato starch (**RPS**), or 50 g/kg high-amylose corn starch (**HCS**), with or without *Eimeria* challenge. On d 13, challenged birds were orally inoculated with mixed oocysts of *E. maxima, E. acervulina*, and *E. tenella*. There were no *Eimeria* × diet interactions for performance traits. During the starter phase (d 0 – 9), birds fed 25 g/kg RPS had higher (*P* = 0.047) weight gain (**WG**) than the control. In the pre-inoculation (d 9 –13) and prepatent (d 13 –16) phases, the WG and final weight gain (**FBW**) were greater (*P* < 0.05) in birds fed 50 g/kg HCS. *Eimeria* challenge decreased (*P* < 0.05) the WG and elevated (*P* < 0.05) the feed conversion ratio (FCR) during acute infection (d 16 – 19) and compensatory growth phases (d 28 – 35). The challenged birds also had shorter villi (*P* < 0.05), deeper crypts (*P* = 0.001), and increased intestinal permeability (*P* < 0.05). No significant diet × infection interaction nor main effect of diets was observed for the AID of amino acids, except for Trp and Cys. Trp had higher (*P* = 0.018) digestibility in birds fed 50 g/kg HCS than the control, and Cys digestibility was greater (*P* = 0.005) in birds fed diets with 25 g/kg RPS or 50 g/kg HCS than the control. However, *Eimeria challenge* depressed (*P* < 0.05) the AID of Ala and that of most indispensable amino acids. In conclusion, dietary RS improved the early growth performance, possibly via modulation of intestinal microbiota and metabolites, inducing a shift in digestion trajectory, but had limited effects post-infection.

## Introduction

Coccidiosis is the most prevalent enteric disease of major economic importance that affects the broiler industry worldwide, with annual global loss of more than $14 billion (Bafundo, 2025). *Eimeria,* a genus of *apicomplexan* parasites, cause coccidiosis in chickens, disrupting the host's homeostasis and decreasing nutrient absorption and utilization. Seven species of *Eimeria* are widely known to affect chickens but *E. necatrix, E. tenella, E. maxima, and E. acervulina* constitute the major global economic challenge due to their pathogenicity and prevalence. They coinfect in most cases and each affects specific anatomical sites (small intestine and ceca, ceca, mid-intestine and upper-intestine, respectively (Shirley, 1986; Attree et al., 2021).

Over the years, different measures have emerged for controlling coccidiosis. However, the use of functional carbohydrates such as resistant starch (**RS**) to improve intestinal health has recently gained more attention. Dietary RS constitute a promising nutritional approach for improving gut homeostasis in poultry during enteric challenge as reported in ducks and weaned pigs (Bhandari et al., 2009; Zhang et al., 2022), as nutrition impacts all aspects of broiler chickens’ responses to *Eimeria* exposure, including susceptibility, protection, pathogenesis, recovery, and compensatory growth (Gómez-Osorio et al., 2021).

RS are starches that escape digestion in the proximal intestinal tract and exert positive effects on the distal part by undergoing fermentation in the ceca to produce beneficial metabolites such as short-chain fatty acids (**SCFA**) and digesta oligosaccharide profile with prebiotic effects (Oluseyifunmi et al., 2024; Lin and Olukosi, 2021). The increased production of SCFA creates a favorable cecal luminal pH for proliferation of beneficial microbes such as *Bifidobacteria* and *Lactobacilli*, which in turn stimulate SCFA synthesis, supporting the tight junction barrier and enterocyte proliferation (Landon et al., 2012).

The existence of *Lactobacillus* at various locations in the gastrointestinal tract improved nutrition utilization, reduced the colonization of the intestines, and the lesions caused by *E. tenella* (Madlala et al., 2021). *Escherichia coli*-induced bone loss had been alleviated in meat ducks fed diet containing 120 g/kg RPS through SCFA production and microbial modulation (Zhang et al., 2022). In addition, feeding weaned piglets with diets containing 70 g/kg raw potato starch (**RPS**) has been reported to reduce postweaning diarrhea with no detrimental effects on growth performance (Bhandari et al., 2009). Dietary RPS at 50 g/kg inclusion reduced the severity of *Salmonella typhimurium* in weaned pigs through modulation of microbial ecosystem and immune status (Yi et al., 2023).

In the absence of enteric infection, a few studies had previously demonstrated negative effects of dietary RS (Regmi et al., 2011; Liu et al., 2020) or non-detrimental effects (Doti et al., 2014; Qin et al., 2019) on growth performance traits such as the weight gain, feed intake and feed efficiency, but these are highly dependent on factors such as the species, age, physiological status of the animals, intricate peculiarity of individual RS, and dietary concentrations. These factors influence RS resistance to pancreatic amylase, fermentability, digestion, and the eventual growth performance response (Oluseyifunmi, 2025; Oluseyifunmi et al., 2024).

The effects of dietary RS during enteric challenge in poultry have only been investigated in very limited studies, as reported in the previous paragraph. In addition, no study has investigated the potential ameliorative effects of resistant starches on phase-specific growth responses, nutrient digestibility, and intestinal integrity in broilers during *Eimeria* challenge. This experiment investigated the impact of dietary inclusion of RS in mitigating the loss in growth performance, apparent ileal amino acid digestibility, and intestinal integrity during mixed *Eimeria* challenge in broiler chickens. It was hypothesized that dietary RS, as a functional dietary fiber, partly alleviates the negative consequences of *Eimeria* challenge on performance, nutrient utilization, and gut integrity in broiler chickens.

## Materials and methods

### Birds and housing

The University of Georgia Institutional Animal Care and Use Committee approved the experimental procedures (IACUC number: A2021-06-006).

### Resistant starches

The raw potato starch used in the experiment was obtained from Danish potatoes using water extraction and procured from Bakers Authority (Maspeth, NY). The high-amylose corn starch (HI-MAIZE ® 260) used was procured from Ingredion (Westchester, IL) and is a high-amylose corn-based non-chemically modified starch. Both resistant starches are native, non-chemically modified starch granules that resist digestion due to the presence of B-type crystallinity and are categorized as type 2 resistant starches.

### Animal housing, diets, and experimental design

The birds were raised in a building containing 48-floor pens (white pine shavings litter, 4.5 × 0.8 m) in a temperature-controlled environment following recommended lighting and temperature regimes for Cobb 500 broiler chickens, with 22 birds per pen from d 0 to 35 d of age. The diets used included a control (corn-SBM diet with zero inclusion of RS) and three additional diets with inclusion of RS: 25 g/kg RPS, 50 g/kg RPS, or 50 g/kg high-amylose corn starch (**HCS**). The levels of RPS and HCS used were informed by previous research (Oluseyifunmi et al., 2024; Oluseyifunmi, 2025). Each diet was administered either with or without *Eimeria* challenge, constituting eight treatment groups arranged as 4 × 2 factorial. A total of 1,056 Cobb 500 male broiler chicks at day old received the experimental diets from day 0 to 35. Each treatment had 6 replicates and 22 birds per replicate. The analyzed chemical composition of the resistant starches was presented earlier (Oluseyifunmi et al., 2024). Feed and water were provided ad libitum throughout the experiment. The diets were presented as mash in the starter and as pellets in the grower and finisher phases, and formulated to meet the nutrient and energy recommendations for Cobb 500 broiler chickens (Cobb-Vantress, 2022). All the diets were isocaloric and isonitrogenous. The diet compositions are presented in Tables 1, 2, and 3 for the starter, grower, and finisher phases, respectively.

**Table 1:** Ingredients and chemical composition (g/kg) of the starter phase (d 0 to 9) diets.

	Starter diet (d 0 - 9)			
Items	Control	25 g/kg	50 g/kg	50 g/kg
Corn	636	599	561	555
Raw potato starch		25	50	
High-amylose corn starch				50
Soybean meal	329	335	343	342
Soybean oil	1.5	7	12.8	19.8
Dicalcium phosphate	18	18	18	18
Limestone	5.9	5.9	5.87	5.87
Sodium bicarbonate	2.0	2.0	2.0	2.0
Vitamin premix^a^	0.9	0.9	0.9	0.9
Trace minerals premix^b^	0.68	0.68	0.68	0.68
DL-Methionine	1.5	1.52	1.5	1.56
L-Lysine⋅HCl	1.52	1.3	1.23	1.33
L-Threonine	0.6	0.6	0.5	0.6
Titanium dioxide	0	0	0	0
Salt (NaCl)	2.3	2.3	2.3	2.3
Choline chloride	0.7	0.7	0.7	0.7
Phytase (Q blue)^c^	0.1	0.1	0.1	0.1
Total	1000	1000	1000	1000
Calculated nutrients and energy				
Crude protein, g/kg	215	214	215	215
ME, kcal/kg	2938	2938	2938	2938
Ca, g/kg	7.9	8.0	8.0	8.0
Available P, g/kg	4.3	4.3	4.3	4.3
Starch	412	414	415	411
Digestible amino acids, g/kg				
Arg	14	14	14	14
His	5.7	5.7	5.7	5.7
Ile	9.1	9.1	9.2	9.2
Leu	19	18	18	18
Lys	13	13	13	13
Met	4.8	4.8	4.8	4.8
Tyr	7.8	7.8	7.8	7.8
Thr	8.6	8.6	8.6	8.6
Met + Cys	8.4	8.3	8.3	8.3

a Vitamin Premix: Supplemented per kg of diet: thiamin mononitrate, 2.4 mg; nicotinic acid, 44 mg; riboflavin, 4.4 mg; D-Ca pantothenate, 12 mg; vitamin B12 (cobalamin), 12.0 g; pyridoxine HCl, 4.7 mg; D-biotin, 0.11 mg; folic acid, 5.5 mg; menadione sodium bisulfite complex, 3.34 mg; choline chloride, 220 mg; cholecalciferol, 27.5 g; transretinyl acetate, 1,892 g; *α-*tocopheryl acetate, 11 mg; ethoxyquin, 125 mg.

b Mineral Premix: Supplemented as per kg of diet: manganese (MnSO_4_.H_2_O), 60 mg; iron (FeSO_4_.7H_2_O), 30 mg; zinc (ZnO), 50 mg; copper (CuSO_4_.5H_2_O), 5 mg; iodine (ethylene diaminedihydroiodide), 0.15 mg; selenium (NaSeO_3_), 0.3 mg.

c Quantum blue phytase: Phytase (AB Vista, Malborough, UK) supplemental dose of 0.1 g/kg to supply 500 FTU/ kg of feed.

**Table 2:** Ingredients and chemical composition (g/kg) of the grower phase (d 9 to 28) diets.

Grower diet (d 9 - 28)				
Items	Control	25 g/kg	50 g/kg	50 g/kg
Corn	680	642	605	598
Raw potato starch		25	50	
High-amylose corn starch				50
Soybean meal	290	297	304	303
Soybean oil	1.2	6.9	12.6	19.6
Dicalcium phosphate	7.5	7.5	7.4	7.5
Limestone	8.77	8.74	8.77	8.72
Sodium bicarbonate	1.5	1.5	1.5	1.5
Vitamin premix^a^	0.9	0.9	0.9	0.9
Trace minerals premix^b^	0.68	0.68	0.68	0.68
DL-Methionine	1.58	1.6	1.61	1.59
L-Lysine⋅HCl	1.59	1.44	1.29	1.35
L-Threonine	0.4	0.4	0.4	0.4
Titanium dioxide	3	3	3	3
Salt (NaCl)	2.5	2.5	2.5	2.5
Choline	0.7	0.7	0.7	0.7
Phytase (Q blue)^c^	0.1	0.1	0.1	0.1
Total	1000	1000	1000	1000
Calculated nutrients and energy				
Protein, g/kg	200	200	200	200
ME, kcal/kg	2988	2988	2988	2988
Ca, g/kg	6.3	6.3	6.3	6.4
Available P, g/kg	2.5	2.5	2.5	2.5
Starch (analyzed)	439	440	441	437
Digestible amino acids, g/kg				
Arg	13	13	13	13
His	5.3	5.3	5.3	5.3
Ile	8.4	8.4	8.5	8.4
Leu	18	17	17	17
Lys	12	12	12	12
Met	4.7	4.7	4.7	4.7
Tyr	7.2	7.2	7.2	7.2
Val	9.4	9.4	9.4	9.4
Met + Cys	8.1	8.0	8.0	8.0
Analyzed nutrient composition, g/kg				
Protein, g/kg	219	211	213	218
GE, kcal/kg	4450	4412	4478	4503
Total starch	454	424	414	407
Resistant starch	12	28	45	24

a Vitamin Premix: Supplemented per kg of diet: thiamin mononitrate, 2.4 mg; nicotinic acid, 44 mg; riboflavin, 4.4 mg; D-Ca pantothenate, 12 mg; vitamin B12 (cobalamin), 12.0 g; pyridoxine HCl, 4.7 mg; D-biotin, 0.11 mg; folic acid, 5.5 mg; menadione sodium bisulfite complex, 3.34 mg; choline chloride, 220 mg; cholecalciferol, 27.5 g; transretinyl acetate, 1,892 g; *α-*tocopheryl acetate, 11 mg; ethoxyquin, 125 mg.

b Mineral Premix: Supplemented as per kg of diet: manganese (MnSO_4_.H_2_O), 60 mg; iron (FeSO_4_.7H_2_O), 30 mg; zinc (ZnO), 50 mg; copper (CuSO_4_.5H_2_O), 5 mg; iodine (ethylene diaminedihydroiodide), 0.15 mg; selenium (NaSeO_3_), 0.3 mg.

c Quantum blue phytase: Phytase (AB Vista, Malborough, UK) supplemental dose of 0.1 g/kg to supply 500 FTU/ kg of feed.

**Table 3:** Ingredients and chemical composition (g/kg) of the grower phase (d 28 to 35) diets.

Finisher diet (d 28 - 35)				
		Potato starch		Hi-Maize starch
Items	Control	25 g/kg	50 g/kg	50 g/kg
Corn	707	670	632	626
Raw potato starch		25	50	
High-amylose corn starch				50
Soybean meal	266	273	280	279
Soybean oil	3	8.9	15	22
Dicalcium phosphate	5.66	5.81	6	6.2
Limestone	8.6	8.5	8.3	8.2
Sodium bicarbonate	2.0	2.0	2.0	2.0
Vitamin premix^a^	0.9	0.9	0.9	0.9
Trace minerals premix^b^	0.68	0.68	0.68	0.68
DL-Methionine	1.37	1.41	1.38	1.4
L-Lysine⋅HCl	1.1	0.9	0.85	0.9
Salt (NaCl)	2.4	2.4	2.4	2.4
Choline	0.7	0.7	0.7	0.7
Phytase (Q blue)^c^	0.1	0.1	0.1	0.1
Total	1000	1000	1000	1000
Calculated nutrients and energy				
Protein, g/kg	190	190	190	190
ME, kcal/kg	3050	3050	3050	3050
Ca, g/kg	5.8	5.7	5.7	5.8
P, g/kg	4.6	4.6	4.6	4.6
Available P, g/kg	2.2	2.2	2.2	2.2
Starch	455	457	458	454
Digestible amino acids, g/kg				
Arg	12	12	12	12
His	5.1	5.1	5.1	5.1
Ile	7.9	8.0	8.0	8.0
Leu	17	17	17	17
Lys	11	11	11	11
Met	4.4	4.4	4.4	4.4
Tyr	6.9	6.9	6.9	6.8
Thr	7.1	7.1	7.1	7.1
Met + Cys	7.6	7.6	7.5	7.5

a Vitamin Premix: Supplemented per kg of diet: thiamin mononitrate, 2.4 mg; nicotinic acid, 44 mg; riboflavin, 4.4 mg; D-Ca pantothenate, 12 mg; vitamin B12 (cobalamin), 12.0 g; pyridoxine HCl, 4.7 mg; D-biotin, 0.11 mg; folic acid, 5.5 mg; menadione sodium bisulfite complex, 3.34 mg; choline chloride, 220 mg; cholecalciferol, 27.5 g; transretinyl acetate, 1,892 g; *α-*tocopheryl acetate, 11 mg; ethoxyquin, 125 mg.

b Mineral Premix: Supplemented as per kg of diet: manganese (MnSO_4_.H_2_O), 60 mg; iron (FeSO_4_.7H_2_O), 30 mg; zinc (ZnO), 50 mg; copper (CuSO_4_.5H_2_O), 5 mg; iodine (ethylene diaminedihydroiodide), 0.15 mg; selenium (NaSeO_3_), 0.3 mg.

c Quantum blue phytase: Phytase (AB Vista, Malborough, UK) supplemental dose of 0.1 g/kg to supply 500 FTU/ kg of feed.

On d 13, the challenge group was orally inoculated with a solution containing 12,500 sporulated oocysts of *E. maxima*, 12,500 sporulated oocysts of *E. tenella*, and 62,500 sporulated oocysts of *E. acervulina* suspended in 1 ml of distilled water, and the non-challenged groups were gavaged with 1 ml of distilled water. On d 19, one bird per pen was euthanized for intestinal permeability assay using FITC-dextran, and on d 20, the ileal digesta were pooled from 3 birds per pen for evaluation of amino acid digestibility, and jejunal tissues were collected for relative mRNA expression and histomorphology.

### Growth performance measurements

Birds and feed were weighed on days 0, 9, 13, 16, 20, 28, and 35 to evaluate the growth performance response at various phases of *Eimeri*a challenge such as the pre-inoculation phase (d 9 −13) prepatent phase (d 13-16, or 0–3 dpi), acute phase (d 17-19, or 4-6 dpi), the recovery (d 19- 27 or 7 −15 dpi) and compensatory growth (d 28-35, or 16 - 22 dpi) phases. Body weight gain (WG), feed intake (FI), and FCR were corrected for mortality.

### Ileal amino acid digestibility

On d 20, three randomly selected birds per pen were euthanized by CO_2_ asphyxiation. The digesta were collected from the distal half of the ileum (from mid ileum up to 2 cm proximal to ileocecal junction) by flushing with distilled water into plastic containers and stored frozen at −20 °C for later processing for evaluation of ileal amino acid digestibility.

### Jejunal histomorphology

Two birds per pen were randomly selected, and approximately 2-cm segments of mid-jejunal tissues of each bird were collected after careful removal of the adhering tissues and flushing out of the digesta using phosphate-buffered saline. The tissues were subsequently fixed in 10 % neutral buffered formalin, dehydrated in ethanol, cleared with xylene, embedded in paraffin wax, and sectioned into 5-μm sections. The sections were further stained with haematoxylin-eosin (H&E) stains and the images were captured with the aid of BZ microscope (BZ-X800; Keyence Inc., Itasca, IL) at 4 × magnification and analyzed using BZ-X800 Analyzer. The villi height (the tip of the villus to the crypt junction) and the crypt depth (depth of the invagination between adjacent villi) were measured for six villi and the corresponding crypts per section, and the villi height-to-crypt depth ratio was determined.

### Quantitative real-time PCR analysis

Mid sections of jejunal tissues were collected from 1 randomly selected bird per pen and snap-frozen in liquid nitrogen and stored at −80 °C prior to RNA extraction. The extraction was done using QiAzol lysis reagent (QIAGEN, Hilden, Germany), and the RNA samples were purified and normalized accordingly using the procedures described by Oluseyifunmi et al. (2024). Approximately 10 μl volume of the RNA was reverse transcribed to cDNA with the aid of a high-capacity cDNA reverse transcription kit (Thermo Fischer Scientific, Waltham) and the RT-PCR carried out in duplicate using StepOnePlus (Applied Biosystems, Carlsbad, CA, USA) with reaction master mix iTaq Universal SYBR Green Supermix (Bio-Rad, Hercules, CA). The fold change was calculated using the 2^−∆∆CT^ method (Livak and Schmittgen, 2001) and the housekeeping gene used were β-actin and glyceraldehyde-3-phosphate dehydrogenase. The primers sequence and their functions (Oluseyifunmi et al., 2024; Oluseyifunmi, 2025) are presented in Table 4.

**Table 4:** List of primers and their functions.

Symbol	Gene name	Function	Forward primer	Reverse primer
β-actin	βeta-actin	Housekeeping gene	ACCGGACTGTTACCAACACC	GACTGCTGCTGACACCTTCA
GAPDH	Glyceraldehyde-3-phosphate dehydrogenase	Housekeeping gene	TGTGACTTCAATGGTGACAGC	GCTATATCCAAACTCATTGTCATACC.
PepT1	Peptide transporter- 1	Peptide transporter	CCCCTGAGGAGGATCACTGTT	CAAAAGACCAGCAGCAAC. GA
*y*+LAT1	*y* + *L* amino acid transporter 1	Cationic amino acid transporter	CAGAAAACCTCAGAGCTCCCTTT	TGAGTACAGAGCCAGCGCAAT
OCLN	Occludin	Tight junction	CTGCTCTGCCTCATCTGCTTCTTC	CCATCCGCCACGTTCTTCACC
CLDN-1	Claudin-1	Tight junction	GGTGAAGAAGATGCGGATGG	CTGGTGTTAACGGGTGTGA
ZO-1	Zona occluden-1	Tight junction	GCCAACTGATGCTGAACCAA	GGGAGAGACAGGACAGGACT
ZO-2	Zona occluden-2	Tight junction	TCAGCAACAGCAAGGTGAAG	GCACCCATGGCAGTAAGGTA
JAM-2	Junctional adhesion molecule 2	Tight junction	AGCCTCAAATGGGATTGGATT	CATCAACTTGCATTCGCTTCA

### Chemical analyses

Oven-dried and ground diets and ileal digesta (0.5 mm sieve) samples were used for chemical analyses following (AOAC, 2012, 2016). Samples were dried at 100 °C for 24 hours for dry matter determination (Method 934.0) and combustion nitrogen analyzer (LECO, St. Joseph, MI) was used to evaluate the N content of the samples. The titanium dioxide analysis was done using the procedures described by Short et al. (1996). Amino acids (AAs) were analyzed following AOAC Method 982.30E (a, b, c). Samples were hydrolyzed with 6 N HCl containing phenol at 110 °C for 24 hours, and AAs were measured using an ion exchange analyzer with ninhydrin post-column derivatization. Chromatograms were detected at 570 and 440 nm and processed using Agilent Open Lab software. Cys and Met were analyzed as cysteic acid and methionine sulfone by oxidation with performic acid–phenol at 0 °C for 16 hours prior to hydrolysis. Tryptophan was measured via alkaline hydrolysis with barium hydroxide at 110 °C for 20 hours, separated by reverse-phase HPLC, and detected using fluorescence to ensure specificity. Megazyme assay kit (K-AMYL) was used for total starch determination and the resistant starch assay was carried out by Eurofins Food Testing, Netherlands, using the AOAC method 2011.25.

### Calculations

All calculations are presented on a dry matter basis. The apparent ileal digestibility of AA in the assay diets was calculated according to the following equation:

$$AID\;\left(\%\right)=1-\left(\frac{ID\times AI}{AD\times II}\right)\times \;100$$

where ID = titanium concentration in the assay diet (% DM), AI = AA content in ileal digesta (% DM), AD = AA content in the assay diet (% DM), and II = titanium concentration in ileal digesta (% DM).

### Statistical analysis

The statistical model for the experiment was:

$$Y_{ijkl}=\mu +\alpha_{i}+\beta_{j\left(i\right)}+\gamma_{k\left(i\right)}+{\left(\beta \gamma \right)}_{jk\left(i\right)}+\varepsilon_{ijkl}$$

where Y*ijk*l is the response variable, μ is the overall mean, *α*_i_ is the random effect of *i*th block, *β*_j_ represent the fixed effect of jth diet within ith treatment group, γ represents the fixed effect of kth infection status within *i*th treatment group. (*β*γ)_jk_ *is the* interaction effect of jth diet and kth infection status within ith treatment group, and the ε*_ijk_*_l_ is the residual error. Data were analyzed using the mixed model procedure of JMP Pro 13.2.0 (SAS Institute Inc., Cary, NC) in a 4 × 2 factorial arrangements. The factors include three RS diets plus corn-soybean meal control diet with or without *Eimeria* challenge. Means with significant differences were separated using Tukey’s HSD, and the significant P-value was set at *P* ≤ 0.05. Main effects are discussed when there are no significant interactions, whereas the simple effects are described in cases of significant interactions.

## Results

### Growth performance

During the starter phase (Table 5), the birds that received 25 g/kg RPS had greater (*P* = 0.047) weight gain (WG) and final body weight (FBW) (*P* = 0.048) than those that received the control diet, whereas the feed intake (FI) and feed conversion ratio (FCR) were not significantly affected by the treatments. During the pre-inoculation phase (d 9 – 13), the WG (*P* = 0.040) and FBW (*P* = 0.023) were higher, along with reduced FCR, (*P* = 0.001) in birds fed 50 g/kg HCS relative to the control diet.

**Table 5:** Growth performance of broiler chickens fed diets with graded levels of resistant starches in the starter phase (d 0-9) and pre-inoculation phase (d 9-13).

	Starter phase (d 0-9)				Pre-inoculation phase (d 9-13)			
Treatments	WG, g	FI, g	FCR	FBW, g	WG, g	FI, g	FCR	FBW, g
Control (0 RS)	160^b^	207	1.29	204^b^	159^b^	220	1.39^a^	361^b^
25 g/kg RPS	175^a^	220	1.26	218^a^	170^ab^	239	1.41^a^	387^ab^
50 g/kg RPS	171^ab^	216	1.27	215^ab^	167^ab^	233	1.40^a^	381^ab^
50 g/kg HCS	173^ab^	214	1.24	217^ab^	181^a^	235	1.30^b^	397^a^
Pooled SEM	3.77	3.73	0.018	3.76	5.37	6.48	0.020	8.26
Probabilities	0.047	0.104	0.226	0.048	0.040	0.233	0.001	0.023

RPS- Raw potato starch, HCS- High-amylose corn starch.

WG- body weight gain, FI- Feed Intake, FCR- feed conversion ratio, FBW: Final Body Weight.

*n* = 12 replicate pens per treatment, with 22 birds per pen.

ab Means in a column with different superscripts differ significantly (*P* ≤ 0.05).

The diet × infection was not significant during the prepatent phase (0–3 dpi) (Table 6). The main effects of diets were significant (*P* < 0.05) for WG, FCR, and FBW, and the main effects of infection (*P* < 0.05) for WG, FI, and FBW. The WG (*P* = 0.001) and FBW (*P* = 0.003) of birds fed 50 g/kg HCS surpassed that of the control diet, coupled with reduced (*P* = 0.015) FCR in the same group of birds. The challenged birds had greater WG (*P* = 0.003), FI (*P* = 0.011), and FBW (*P* < 0.001) than the unchallenged birds in the prepatent phase of infection.

**Table 6:** Growth performance of broiler chickens fed diets with graded levels of resistant starches during the prepatent (0 - 3 dpi) and acute (4 - 6 dpi) phases when challenged or unchallenged with mixed *Eimeria* spp. oocysts.

			Prepatent phase (d 13-16 or 0-3 dpi)				Acute phase (d 17-19 or 4-6 dpi)			
Items		Treatments	WG, g	FI, g	FCR	FBW, g	WG, g	FI, g	FCR	FBW, g
Diet × Infection			Simple effect means							
	Infected	Control (0 RS)	176	260	1.48	543	89	2	2.62	621
		25 g/kg RPS	188	262	1.40	599	87	229	2.63	673
		50 g/kg RPS	186	260	1.40	581	75	216	2.88	656
		50 g/kg HCS	197	264	1.34	608	57	212	3.72	660
	Uninfected	Control (0 RS)	169	242	1.44	523	222	323	1.45	746
		25 g/kg RPS	176	241	1.37	539	222	328	1.48	760
		50 g/kg RPS	174	250	1.44	541	223	322	1.44	765
		50 g/kg HCS	187	257	1.38	571	239	321	1.34	810
			Main effect means							
Diets		Control (0 RS)	172^b^	251	1.46^a^	533^b^	155	279	2.04	693
		25 g/kg RPS	182^ab^	252	1.39^ab^	569^ab^	154	278	2.03	723
		50 g/kg RPS	180^ab^	255	1.42^ab^	561^ab^	149	269	2.16	711
		50 g/kg HCS	192^a^	261	1.36^b^	589^a^	148	267	2.53	735
			Main effect means							
Infection status		Infected	187	262	1.40	583	77	223	2.96	660
		Uninfected	176	248	1.41	544	226	323	1.43	770
			Pooled SEM							
		Diets	3.26	5.19	0.022	9.77	10.98	9.83	0.439	16.67
		Infection	2.30	3.67	0.016	6.91	7.77	6.96	0.311	12.50
		Diet × Infection	4.61	7.35	0.031	13.81	15.30	14.81	0.620	24.99
			Probabilities							
		Diets	0.001	0.528	0.015	0.003	0.911	0.692	0.384	0.454
		Infection	0.003	0.011	0.823	< 0.001	< 0.001	< 0.001	< 0.001	< 0.001
		Diet × Infection	0.946	0.744	0.583	0.569	0.302	0.850	0.279	0.363

RPS- Raw potato starch, HCS- High-amylose corn starch.

WG- body weight gain, FI- Feed Intake, FCR- feed conversion ratio, FBW: Final Body Weight.

*n* = 6 replicate pens per treatment for simple effects, *n* = 12 replicate pens per treatment for main effects of diets, *n* = 24 replicate pens per treatment for main effects of infection status, with 22 birds per pen.

Prepatent phase (0–3 dpi), acute phase (4-6 dpi), dpi- days post-inoculation.

The birds in the infected group were inoculated with mixed oocysts of *E. maxima (12,500), E. tenella (12,500), E. acervulina (62,500)* per ml on d 13 of age. The non-infected group received oral gavage of 1 ml of distilled water.

ab Means in a column, within a group, with different superscripts differ significantly (*P* ≤ 0.05).

During the acute phase (4-6 dpi), there were no significant diet × infection nor main effects of diet on the growth performance responses, except for the significant (*P* < 0.05) main effects of infection (Table 6). The *Eimeria* challenge reduced (*P* < 0.001) the WG, FI, and FBW and increased (*P* < 0.001) the FCR of the birds, irrespective of the diets fed.

Neither the diet × infection nor the main effects of diets were significant during the recovery (7 −15 dpi) and compensatory growth (16 - 22 dpi) phases (Table 7). The main effects of infection were significant (*P* < 0.05) for the WG, FI, and FBW during the recovery phase and the WG, FCR, and FBW during the compensatory growth phase. The growth responses were negatively impacted in the birds challenged with *Eimeria* during the recovery and compensatory growth phases, marked by reduced WG (*P* = 0.023), FI and FBW (*P* < 0.001) in the recovery phase and decreased WG (*P* = 0.003), and FBW (*P* = 0.001) along with increased FCR (*P* = 0.002) in the compensatory growth phase.

**Table 7:** Growth performance of broiler chickens fed diets with graded levels of resistant starches during the recovery (7 - 15 dpi) and compensatory (16 - 22 dpi) phases when challenged or unchallenged with mixed *Eimeria* spp. oocysts.

			Recovery phase (d 19-27, or 7-15 dpi)				Compensatory phase (d 28-35, or 16-22 dpi)			
Items		Treatments	WG, g	FI, g	FCR	FBW, g	WG, g	FI, g	FCR	FBW, g
Diet × Infection			Simple effect means							
	Infected	Control (0 RS)	772	1180	1.55	1393	834	1403	1.71	2262
		25 g/kg RPS	807	1202	1.49	1479	872	1424	1.64	2392
		50 g/kg RPS	815	1238	1.55	1471	882	1464	1.68	2391
		50 g/kg HCS	814	1173	1.44	1465	828	1422	1.72	2331
	Uninfected	Control (0 RS)	845	1340	1.59	1591	952	1449	1.52	2584
		25 g/kg RPS	863	1342	1.56	1623	956	1478	1.55	2618
		50 g/kg RPS	865	1369	1.62	1632	983	1491	1.53	2652
		50 g/kg HCS	906	1352	1.54	1715	882	1428	1.63	2629
			Main effect means							
Diets		Control (0 RS)	808	1260	1.57	1492	893	1426	1.61	2423
		25 g/kg RPS	835	1272	1.52	1551	914	1451	1.60	2505
		50 g/kg RPS	840	1303	1.58	1552	933	1478	1.60	2521
		50 g/kg HCS	860	1262	1.49	1590	855	1425	1.68	2480
			Main effect means							
Infection status		Infected	802	1198	1.51	1452	854	1429	1.69	2344
		Uninfected	870	1351	1.58	1640	943	1462	1.56	2621
			Pooled SEM							
		Diets	28.82	21.98	0.063	32.77	27.72	23.20	0.039	44.76
		Infection	20.38	15.54	0.045	23.17	19.60	16.40	0.027	31.65
		Diet × Infection	40.76	31.08	0.089	46.34	39.20	32.80	0.055	63.30
			Probabilities							
		Diets	0.653	0.490	0.723	0.224	0.242	0.340	0.488	0.437
		Infection	0.023	< 0.001	0.295	< 0.001	0.003	0.163	0.002	< 0.001
		Diet × Infection	0.955	0.869	0.987	0.675	0.863	0.894	0.783	0.881

RPS- Raw potato starch, HCS- High-amylose corn starch.

WG- body weight gain, FI- Feed Intake, FCR- feed conversion ratio, FBW: Final Body Weight.

*n* = 6 replicate pens per treatment for simple effects, *n* = 12 replicate pens per treatment for main effects of diets, *n* = 24 replicate pens per treatment for main effects of infection status, with 22 birds per pen.

Recovery (7 −15 dpi) and compensatory growth (16 - 22 dpi) phases, dpi- days post-inoculation.

The birds in the infected group were inoculated with mixed oocysts of *E. maxima (12,500), E. tenella (12,500), E. acervulina (62,500)* per ml on d 13 of age. The non-infected group received oral gavage of 1 ml of distilled water.

### Apparent ileal amino acid digestibility -indispensable amino acids

There was no significant diet × infection interaction or main effects of diet for dry matter digestibility (DMD) or AID of indispensable amino acids, except for Trp (Table 8). Birds fed 50 g/kg HCS had greater (*P* = 0.018) AID of Trp than those that received the control diet. The main effects of infection were significant (*P* < 0.05) for the AID of His, Ile, Lys, Met, Phe, Val, and Trp. The AID of His (*P* = 0.046), Ile (*P* = 0.025), Lys (*P* = 0.008), Met (*P* = 0.002), Phe (*P* = 0.034), and Val (*P* = 0.016) were reduced in the challenged birds, except for the AID of Trp that was greater (*P* < 0.001).

**Table 8:** Apparent ileal digestibility of indispensable amino acid in broiler chickens fed diets with graded levels of resistant starches at d 20 (during recovery phase) when challenged or unchallenged with mixed *Eimeria* spp. oocysts.

Items		Treatments	DMD	Arg	His	Ile	Leu	Lys	Met	Phe	Thr	Val	Trp
Diet × Infection			Simple effect means										
	Infected	Control (0 RS)	66.3	83.5	75.5	72.5	74.6	75.6	81.7	74.6	65.4	70.3	82.6
		25 g/kg RPS	67.4	85.5	77.3	74.8	76.3	77.8	82.7	76.6	67.7	72.5	84.2
		50 g/kg RPS	64.2	84.4	76.1	73.4	75.4	76.0	80.9	75.6	64.9	71.0	84.0
		50 g/kg HCS	63.1	83.4	74.8	72.8	74.4	76.7	81.6	75.1	66.3	70.3	84.8
	Uninfected	Control (0 RS)	65.5	82.3	75.3	72.9	73.1	77.0	82.5	74.8	65.4	71.0	78.3
		25 g/kg RPS	67.1	84.9	77.8	75.9	76.2	79.2	84.0	77.7	67.3	73.8	79.2
		50 g/kg RPS	66.7	84.3	77.4	75.3	75.6	78.5	83.4	77.0	66.5	73.2	78.1
		50 g/kg HCS	68.2	86.4	80.2	78.3	78.6	82.7	87.7	79.8	71.4	76.6	83.2
			Main effect means										
Diets		Control (0 RS)	65.9	84.9	75.4	72.7	73.9	76.3	82.1	74.7	65.4	70.7	80.4^b^
		25 g/kg RPS	67.3	86.3	77.6	75.3	76.3	78.5	83.3	77.1	67.5	73.2	81.7^ab^
		50 g/kg RPS	65.4	86.4	76.8	74.3	75.5	77.2	82.1	76.3	65.7	72.1	81.0^ab^
		50 g/kg HCS	65.7	86.8	77.5	75.6	76.5	79.7	84.7	77.4	68.9	73.5	84.0^a^
Infection status			Main effect means										
		Infected	65.3	84.2	75.9	73.4	75.2	76.5	81.7	75.5	66.1	71.1	83.9
		Uninfected	66.9	84.5	77.7	75.6	75.9	79.4	84.4	77.3	67.6	73.7	79.7
			Pooled SEM										
		Diets	0.979	0.676	0.842	0.947	0.922	1.003	0.567	0.849	1.214	1.022	0.793
		Infection	0.692	0.478	0.595	0.670	0.652	0.709	0.401	0.600	0.858	0.723	0.561
		Diet × Infection	1.385	0.956	1.191	1.339	1.304	1.42	0.802	1.2006	1.72	1.445	1.122
			Probabilities										
		Diets	0.546	0.110	0.247	0.146	0.195	0.101	0.101	0.120	0.164	0.226	0.018
		Infection	0.104	0.696	0.046	0.025	0.454	0.008	0.002	0.034	0.207	0.016	< 0.001
		Diet × Infection	0.131	0.156	0.105	0.246	0.180	0.339	0.115	0.287	0.380	0.231	0.276

RPS- Raw potato starch, HCS- High-amylose corn starch, DMD- Dry matter digestibility.

*n* = 6 replicate pens per treatment for simple effects, *n* = 12 replicate pens per treatment for main effects of diets, *n* = 24 replicate pens per treatment for main effects of infection status, with 22 birds per pen.

Recovery phase (7- 15 dpi), dpi- days post-inoculation.

The birds in the infected group were inoculated with mixed oocysts of *E. maxima (12,500), E. tenella (12,500), E. acervulina (62,500)* per ml on d 13 of age. The non-infected group received oral gavage of 1 ml of distilled water.

ab : Means in a column, within a group, with different superscripts differ significantly (*P* ≤ 0.05).

### Apparent ileal amino acid digestibility - dispensable amino acids

There was no significant diet × infection for AID of all the dispensable amino acids assessed (Table 9). However, the main effect of diets was significant (*P* < 0.05) for Cys (*P* **<**0.05**)** and showed a tendency (*P* < 0.10) for Asp, Gly, Glu and Ser. The birds that received 25 g/kg RPS and 50 g/kg HCS diets had greater (*P* = 0.005) AID of Cys than those that received the control diet. The AID of Asp, Gly, Glu, and Ser tended (*P* < 0.10) to increase in birds fed 25 g/kg RPS and 50 g/kg HCS diets. Additionally, the main effect of *Eimeria* infection was significant (*P* < 0.05) for AID of Ala and showed a tendency for AID of Cys. The AID of Ala decreased (*P* = 0.002) in the challenged birds, and that of Cys tended (*P* = 0.077) to decrease during infection as well.

**Table 9:** Apparent ileal digestibility of dispensable amino acid in broiler chickens fed diets with graded levels of resistant starches on d 20 (during recovery phase) when challenged or unchallenged with mixed *Eimeria* spp. oocysts.

Items		Treatments	Ala	Asp	Cys	Gly	Glu	Pro	Ser	Tyr
Diet × Infection			Simple effect means							
	Infected	Control (0 RS)	70.5	73.6	59.5	67.8	81.3	73.8	73.6	75.4
		25 g/kg RPS	72.5	76.0	66.9	70.9	83.1	76.1	76.2	77.4
		50 g/kg RPS	70.6	74.6	61.2	68.8	82.1	74.4	74.1	76.6
		50 g/kg HCS	69.8	74.7	61.2	68.9	81.5	73.6	74.8	75.8
	Uninfected	Control (0 RS)	71.7	73.4	54.5	66.4	79.8	71.4	71.9	75.0
		25 g/kg RPS	74.7	76.5	61.8	70.1	82.7	75.0	74.6	77.4
		50 g/kg RPS	73.7	75.5	57.7	68.7	82.0	73.7	73.4	77.1
		50 g/kg HCS	77.2	78.0	64.3	73.0	84.3	76.4	77.4	79.0
			Main effect means							
Diets		Control (0 RS)	71.1	73.5	57.01^b^	67.1	80.6	72.6	72.7	75.2
		25 g/kg RPS	73.6	76.3	64.37^a^	70.5	82.9	75.5	75.4	77.4
		50 g/kg RPS	72.1	75.1	59.45^ab^	68.8	82.0	74.0	73.8	76.8
		50 g/kg HCS	73.5	76.3	62.76^a^	70.9	82.9	75.0	76.1	77.4
Infection status			Main effect means							
		Infected	70.8	74.7	62.2	69.1	82.0	74.5	74.7	76.3
		Uninfected	74.3	75.9	59.6	69.5	82.2	74.1	74.3	77.1
			Pooled SEM							
		Diets	1.027	0.872	1.461	1.095	0.682	0.895	0.931	0.967
		Infection	0.726	0.616	1.033	0.774	0.482	0.633	0.658	0.684
		Diet × Infection	1.452	1.233	2.065	1.548	0.964	1.266	1.316	1.368
			Probabilities							
		Diets	0.263	0.090	0.005	0.073	0.069	0.128	0.061	0.348
		Infection	0.002	0.205	0.077	0.701	0.780	0.708	0.740	0.393
		Diet × Infection	0.177	0.531	0.175	0.289	0.169	0.206	0.334	0.582

RPS- Raw potato starch, HCS- High-amylose corn starch.

*n* = 6 replicate pens per treatment for simple effects, *n* = 12 replicate pens per treatment for main effects of diets, *n* = 24 replicate pens per treatment for main effects of infection status, with 22 birds per pen.

Recovery phase (7- 15 dpi), dpi- days post-inoculation.

The birds in the infected group were inoculated with mixed oocysts of *E. maxima (12,500), E. tenella (12,500), E. acervulina (62,500)* per ml on d 13 of age. The non-infected group received oral gavage of 1 ml of distilled water.

ab : Means in a column, within a group, with different superscripts differ significantly (*P* ≤ 0.05).

### Gastrointestinal tract permeability

**Fig. 1:**
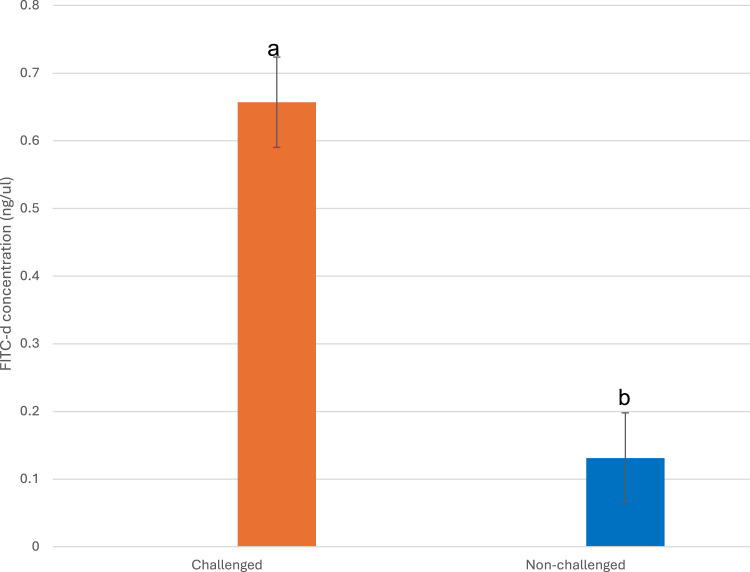
The intestinal permeability on day 19 (6 dpi). The *Eimeria*-challenged birds had significantly higher (*P* < 0.001) serum levels of FITC-d. The birds in the infected group were inoculated with mixed oocysts of *E. maxima* (12,500), *E. tenella* (12,500), *E. acervulina* (62,500) per ml on d 13 of age. The non-infected group received oral gavage of 1 ml of distilled water.

The gut permeability assessed on d 19 (6 dpi) (Fig. 1) was not significantly (*P* > 0.05) affected by the interaction of diet × infection or main effects of diets but the main effect of infection was significant (*P* < 0.05). The *Eimeria*-challenged birds had greater (*P* < 0.001) serum levels of FITC-d than the unchallenged group.

### Jejunal histomorphology

The histomorphology results showed no significant (*P* > 0.05) interaction of diet × challenge or main effects of diets (Table 10). However, the *Eimeria* challenge reduced the jejunal VH (*P* < 0.001), VH:CD (*P* < 0.001), and increased (*P* = 0.001) the CD.

**Table 10:** Jejunal histomorphology on d 20 (during recovery phase) in broiler chickens fed diets with graded levels of resistant starches when challenged or unchallenged with mixed *Eimeria* spp. oocysts.

Items		Treatments	VH (µm)	CD (µm)	VW (µm)	VH/CD
Diet × Infection			Simple effect means			
	Infected	Control (0 RS)	745	252	260	3.07
		25 g/kg RPS	779	267	225	3.16
		50 g/kg RPS	789	281	247	2.87
		50 g/kg HCS	770	285	263	2.73
	Uninfected	Control (0 RS)	1215	217	242	5.76
		25 g/kg RPS	1379	203	236	6.82
		50 g/kg RPS	1304	241	249	5.55
		50 g/kg HCS	1509	218	255	6.92
			Main effect means			
Diets		Control (0 RS)	980	234	251	4.41
		25 g/kg RPS	1079	235	230	4.99
		50 g/kg RPS	1046	261	248	4.21
		50 g/kg HCS	1140	251	259	4.83
			Main effect means			
Infection status		Infected	771	271	249	2.96
		Uninfected	1352	220	246	6.26
			Pooled SEM			
		Diets	61.36	14.62	11.24	0.304
		Infection	43.49	10.34	7.95	0.215
		Diet × Infection	84.71	20.67	15.89	0.430
			Probabilities			
		Diets	0.330	0.493	0.361	0.257
		Infection	< 0.001	0.001	0.791	< 0.001
		Diet × Infection	0.444	0.821	0.807	0.227

RPS- Raw potato starch, HCS- High-amylose corn starch.

VH- Villi height. CD- Crypt depth, VW- Villi width.

*n* = 6 replicate pens per treatment for simple effects, *n* = 12 replicate pens per treatment for main effects of diets, *n* = 24 replicate pens per treatment for main effects of infection status, with 22 birds per pen.

Recovery phase (7- 15 dpi), dpi- days post-inoculation.

The birds in the infected group were inoculated with mixed oocysts of *E. maxima (12,500), E. tenella (12,500), E. acervulina (62,500)* per ml on d 13 of age. The non-infected group received oral gavage of 1 ml of distilled water.

### Relative mRNA expression of selected jejunal tight junction proteins and nutrient transporters

The relative mRNA expressions of jejunal tight junction proteins were not significantly affected by the interaction of the diet × infection and main effects of diets, except for the main effects of infection on JAM-2 and a tendency (*P* < 0.10) for occludin (Table 11). The *Eimeria* challenge reduced (*P* = 0.001) the expression of JAM-2 and tended (*P* = 0.069) to reduce the expression of occludin. The diet × infection was significant (*P* = 0.054) for PepT1, which had greater expression in unchallenged birds fed 25 g/kg RPS relative to challenged birds fed 25 and 50 g/kg RPS. Among the challenged birds, those fed the control or 50 g/kg HCS had greater relative PepT1 expression.

**Table 11:** Relative mRNA expression of selected jejunal tight junction proteins and nutrient transporters on d 20 (during recovery phase) in broiler chickens fed diets with graded levels of resistant starches when challenged or unchallenged with mixed *Eimeria* spp. oocysts.

Items		Treatments	ZO-1	ZO-2	JAM-2	Claudin	Occludin	Pept1	*y*+LAT
Diet × Infection			Simple effect means						
	Infected	Control (0 RS)	1.34	0.39	0.45	0.92	1.34	0.92^ab^	0.68
		25 g/kg RPS	0.63	0.78	0.50	1.29	0.63	0.36^bc^	0.60
		50 g/kg RPS	0.35	0.98	0.14	0.21	0.35	0.57^b^	1.42
		50 g/kg HCS	1.17	0.89	0.48	1.48	1.17	0.79^ab^	0.27
	Uninfected	Control (0 RS)	1.00	1.00	1.00	1.00	1.00	1.00^ab^	1.00
		25 g/kg RPS	1.60	0.33	0.96	0.80	1.60	1.57^a^	0.87
		50 g/kg RPS	1.54	0.63	1.14	1.35	1.54	0.84^ab^	1.35
		50 g/kg HCS	1.83	0.50	1.44	1.02	1.83	1.14^ab^	0.91
			Main effect means						
Diets		Control (0 RS)	1.17	0.70	0.73	0.96	1.17	0.96	0.84
		25 g/kg RPS	1.11	0.56	0.73	1.04	1.11	0.97	0.74
		50 g/kg RPS	0.94	0.80	0.64	0.78	0.94	0.71	1.38
		50 g/kg HCS	1.50	0.69	0.96	1.25	1.50	0.96	0.59
Infection status			Main effect means						
		Infected	0.87	0.76	0.39	0.97	0.87	0.76	0.74
		Uninfected	1.49	0.62	1.14	1.04	1.49	0.99	1.03
			Pooled SEM						
		Diets	0.234	0.183	0.194	0.357	0.342	0.192	0.385
		Infection	0.167	0.130	0.137	0.252	0.243	0.135	0.277
		Diet × Infection	0.331	0.259	0.277	0.505	0.482	0.271	0.539
			Probabilities						
		Diets	0.647	0.832	0.735	0.814	0.698	0.309	0.808
		Infection	0.329	0.348	0.001	0.869	0.069	0.059	0.800
		Diet × Infection	0.845	0.150	0.668	0.295	0.410	0.054	0.905

RPS- Raw potato starch, HCS- High-amylose corn starch.2.

*n* = 6 replicate pens per treatment for simple effects, *n* = 12 replicate pens per treatment for main effects of diets, *n* = 24 replicate pens per treatment for main effects of infection status, with 22 birds per pen.

^abc^^:^ Means in a column, within a group, with different superscripts differ significantly (*P* ≤ 0.05). *n* = 6 replicate cages per treatment.

Recovery phase (7- 15 dpi), dpi- days post-inoculation.

The birds in the infected group were inoculated with mixed oocysts of *E. maxima (12,500), E. tenella (12,500), E. acervulina (62,500)* per ml on d 13 of age. The non-infected group received oral gavage of 1 ml of distilled water.

## Discussion

Over the years, the modulation of dietary compositions and formulations has constituted part of the major control measures for coccidiosis (Gómez-Osorio et al., 2021). Dietary resistant starches have gained attention in recent times for their potential to improve gut health and mitigate the negative impacts of enteric challenge, owing to their prebiotic properties and ability to modulate the hindgut microbial ecology (Thompson et al., 2022). This study investigated the possible prebiotic effects of resistant starches in broiler chickens during enteric challenge. We therefore hypothesized a phase-specific dietary RS modulation of the growth responses in broiler chicken challenged with mixed *Eimeria* spp. The choice of the RS and levels used in the current experiment were based on previous experiments (Oluseyifumi et al., 2024; Oluseyifunmi, 2025).

### Growth performance

The results obtained in this study indicated greater WG in birds fed diets with inclusion of 25 g/kg RPS during the starter phase and 50 g/kg HCS during the early grower phase (pre-inoculation phase). This showed a phase-dependent impact of resistant starch (RS) on growth performance, suggesting that different RS sources and concentrations might influence differently at different phases of growth in broiler chicken.

Liu et al. (2020) previously reported a lower weight gain and feed intake and a decline in feed efficiency as the dietary RS level increased in chickens fed diets containing 40, 80, and 120 g/kg corn RS relative to the control diet (corn-soybean-based diet with 200 g/kg corn starch). Zhang et al. (2020) further compared the same set of diets with corn-soybean-based control diet in broiler chickens and found lower feed intake and weight gain in the birds that received RS diets and the diet containing 200 g/kg corn starch.

On the contrary, feeding day-old duckling diets with diets containing 60, 120, 180, and 240 g/kg RPS for a period of 14 and 35 days had no significant adverse effects on the growth performance relative to the corn-soybean-based control diet (Qin et al., 2019). The variation in results could be attributed to different botanical origins of RS, varying dietary RS content, and experimental models (ducks vs. broilers), feeding duration, and whether the birds were challenged or unchallenged with enteric pathogens) in these studies. Lower concentrations of RS were used in the current experiment because higher inclusion levels (up to 100 g/kg) had been previously tested but were found to be difficult to incorporate in pelleted diets resulting in pasty pellets, as well as such diets are of little practical relevance from economics standpoint because of availability of only food-grade RS (Oluseyifunmi et al., 2024). The increase in WG observed in the current study can be explained by the positive effects of RPS and HCS on nitrogen retention and energy utilization, as demonstrated in our previous study (Oluseyifunmi et al., 2024).

The higher feed intake and weight gain observed at the early stage of infection (prepatent phase) might be because the infection is yet to peak at this time point, and the pathological consequence of coccidiosis might still be less severe during the incubation stage (de Freitas et al., 2023). In addition, the continuous supply of energy through production of SCFA by fermentation of RS in distal digestive tract in birds fed ad libitum, coupled with the modulation of nitrogen and energy metabolism by HCS (Oluseyifunmi et al., 2024) might have also played a role in alleviating the effects of coccidiosis at this phase of infection.

Coccidiosis had detrimental effects on the WG, FI and FCR during the acute and compensatory growth phases with no significant ameliorative impacts of RS observed during these phases. This can be explained by repartitioning of nutrients towards intestinal repair, immune responses, and disruption of digestive and absorptive capacity, which have been reported to account for about 28 % reduction in body weight and 73 % decrease in body weight as consequences of reduced FI during coccidiosis (Lillehoj and Trout, 1996; Lee and Rochell, 2022).

### Apparent ileal amino acid digestibility

The AID value indicates the net disappearance of amino acids from the digestive tract (Kim et al., 2022). The effects of dietary fibers on nitrogen and amino acid digestibility are dependent on several factors, including the nature of the fiber, digestibility of dietary carbohydrate and protein, and the physiological condition of the animals. Native fibers may play a role in the sloughing of intestinal cells and have the potential to adsorb amino acids, peptides, or proteins (Eggum, 1992).

*Eimeria* challenge could impair the digestive and absorptive capacity of the gastrointestinal tract and interfere with apparent ileal digestibility of amino acids and growth performance (Rochell et al., 2016a). In the current experiment, *Eimeria* infection reduced the AID of selected indispensable AA which included Met and Lys, the most limiting amino acids for broiler growth performance (Alagawany et al., 2021). These amino acids are important for combating oxidative stress and crucial for immune functions (Jespersen et al., 2024). The AID of Ala was also reduced by mixed *Eimeria* challenge irrespective of the dietary RS fed. The digestibility of Ala has been reported previously to be one of the most impacted AA during mixed *Eimeria* challenge model (Kim et al., 2022)*.*

The observed decrease in the AID of these AA in the infected birds could be a consequence of disruption of gut structure (villus atrophy, crypt hyperplasia, thinning of mucosa) and function (reduced absorption and digestive enzyme activity) thus making the animal to excrete more (Lee and Rochell, 2022). Another probable reason might be the birds’ innate response to reduce AA absorption in order to “starve” the infectious organism, as demonstrated with bacterial infection which may trigger the intracellular induction of AA starvation in order to reduce nutrient availability to the infectious organism (Tattoli et al., 2012). In addition, it has been reported that *Eimeria* challenge may reduce AA digestibility by an average of 8 % relative to the unchallenged birds (Rochell et al., 2016b; Adedokun et al., 2016; Lee and Rochell, 2022).

Tryptophan plays an important role in modulating gut microbiota as it serves as a substrate for enzymes within the gut microbiota, leading to its breakdown into various metabolites, which can regulate the host's immune response (Gao et al., 2020). Therefore, the elevated AID of Trp during the mixed *Eimeria* challenge in this study is not unexpected because Trp is involved in acute protein synthesis during inflammation, and birds may increase their requirement for it during *Eimeria* challenge (Kim et al., 2022). Although Rochell et al. (2016b) previously reported that *E. acervulina* did not affect the AID of Trp and Gly among other AA, the current study utilized a mixture of *E. maxima, E. tenella*, and *E. acervulina;* thus the enteric challenge is expected to be more severe as each affects different and specific anatomic sites*.*

The increase in the AID of Trp in the birds fed diet containing 50 g/kg HCS and greater AID of Cys in the birds that received diets with 25 g/kg RPS and 50 g/kg HCS indicated possible modulation of AA metabolism by dietary inclusion of RS as previously reported by Qin et al. (2023), although the mechanism behind this is yet to be understood. Resistant starches modulate microbial ecology towards beneficial profile (Tan et al., 2021) and evidence exists that some bacteria species may secrete dipeptidyl peptidase and dipeptidase that contribute to protein digestion and absorption of amino acids in the gut (Torres et al., 2023). In addition, starches that supply the lower part of the intestine with glucose may have some sparing effects on amino acids, preventing them from being oxidized (Weurding et al., 2003).

### Gastrointestinal tract permeability

The intestinal permeability is commonly measured in poultry using fluorescein isothiocyanate-dextran (FITC-d) as an indicator (Liu et al., 2021). The elevated FITC-d levels in the serum of the infected birds on d 19 (6 dpi) points to the severity of *Eimeria* challenge at the peak of infection (acute phase) and is indicative of the elevated leakage of the gastrointestinal tract that eventuated from invasion of the host epithelial cells by merozoites during asexual reproduction (Lin and Olukosi, 2021). Coccidiosis causes the disruption of the mucosal and epithelial barrier, leading to inflammation characterized by vasodilation and increased intestinal permeability (Lee and Rochell, 2022).

However, dietary inclusion of RS had no ameliorative effect on gut leakage during the *Eimeria* challenge model used in the current study. This could be due to the localization of RS activity in the distal gastrointestinal tract, where they are fermented to produce beneficial metabolites such as butyrate, propionate, and acetate, which can support intestinal integrity and possess anti-inflammatory properties (Tan et al., 2021). In addition, the extent of intestinal tissue disruption in the current experiment may be beyond the ameliorative capacity of the RS used due to the observation that the extent of intestinal tissue disruption is proportional to the concentration of oocysts used in the challenge (Teng et al., 2021).

### Jejunal histomorphology

Villi height to crypt depth ratio is an indicator of intestinal absorptive capacity and health (Marchewka et al., 2021). The reduced VH and VH:CD are indicative of villous atrophy, decreasing absorptive surface area as evidenced by the reduced apparent ileal digestibility of most of the AA evaluated in the challenged birds (Belote et al., 2023; Liu et al., 2023; Ajao et al., 2024; Taylor et al., 2024). The hyperplasia of jejunal crypts observed in *Eimeria*-challenged birds suggested increased proliferative activity of crypt cells, which are higher during inflammation (Bortoluzzi et al., 2020).

Although a previous work by Qin et al. (2020), reported improvements in VH, VH:CD, and reduced CD in the cecum of ducks fed high levels of raw potato starch (120 and 240 g/kg RPS-containing diets compared to those fed 0 % RPS for a period of 35 days). The RS inclusion did not yield similar benefits in the jejunum under our enteric challenge model likely because of the differences in the two experiments based on the species of birds, digestive site, and level of RS inclusion. These differences may be further explained by the severity of the acute *Eimeria* infection which could have masked the potential ameliorative effects of RS, the relatively short feeding duration, or the site-specific action of RS, as its fermentation primarily occurs in the distal gut and may not sufficiently impact jejunal morphology during peak infection.

### Relative mRNA expression of selected jejunal tight junction proteins and nutrient transporters

Tight junction proteins are complex transmembrane proteins crucial for maintaining the functionality and integrity of intestinal epithelial cell barriers by forming tight seals that control the transcellular and paracellular translocations of molecules and ions, preventing pathogen entry (Graham et al., 2023). The depression of relative mRNA expression of JAM-2 and occludin observed during mixed *Eimeria* challenge in the current study is indicative of the impairment of the tight junction barrier due to sloughing of the intestinal epithelial cells and disruption of the mucus layer during coccidiosis. Similar findings were reported by (Scharl et al., 2009; Lin et al., 2022) in *Eimeria*-challenged birds. *Eimeria* challenge may alter the gene expression of tight junctions, adhering junctions, gap junctions and desmosomes due to sloughing and impairment of the intestinal mucosa along with vasodilation of intestinal epithelium in response to enteric inflammation (Lee and Rochell, 2022).

PepT1 is a protein situated in the brush-border membranes that facilitates the transport of di- and tripeptides (Verri et al., 2010). *Eimeria* challenge may downregulate the AA transporters and peptides, along with brush border digestive enzymes as a part of the defense mechanism against infectious organism replication (Lin et al., 2022, 2023) Lee and Rochell, 2022; Lin et al., 2023). Greater relative mRNA expression of PepT1 during infection in the birds fed diet with 50 g/kg HCS than in other infected birds that received other RS diets demonstrated the potential of HCS in facilitating the luminal uptake of peptides during enteric challenge in broiler chickens. Additionally, a much higher expression of PepT1 in birds fed dietary 25 g/kg RPS and 50 g/kg HCS in the absence of *Eimeria* challenge is consistent with the previous report (Oluseyifunmi et al., 2024). Modulation of intestinal microbiota by RS fermentation metabolite (SCFA) may favor proliferation of beneficial bacteria, which could secrete some dipeptidyl peptidase and dipeptidase that may influence protein digestion and absorption of amino acids in the gut, although this has not been established in chickens (Tan et al., 2021; Tores et al., 2023).

## Conclusion

Dietary inclusion of 25 g/kg RPS and 50 g/kg HCS improved the growth performance during the early growth phase until the acute phase of *Eimeria* infection and therefore showed limited significant positive effects during the subsequent phases, consistent with the effect on AA digestibility. Dietary RS improved the early growth performance, possibly via modulation of intestinal microbiota and shift in digestion trajectory, but the limited effects post-infection may be challenge-dose-dependent. The observations of the current experiment indicated potentially limited ameliorative effects of RS in *Eimeria*-challenged birds and suggested that the pathways by which this is effected are via effect on AA utilization and gut integrity, achievable by possible modulation of gut microbiome and their metabolites, which are subjects of follow-up investigation.

## Funding

This study was sponsored by cooperative agreement 58-6040-8-034 (United States Department of Agriculture) – Agricultural Research Service and partly funded by the Nigeria Tertiary Education Trust Fund (TETFund).

## CRediT authorship contribution statement

**Iyabo W. Oluseyifunmi:** Writing – original draft, Methodology, Investigation, Formal analysis. **Oluyinka A. Olukosi:** Writing – review & editing, Validation, Supervision, Resources, Project administration, Investigation, Funding acquisition, Data curation, Conceptualization.

## Disclosures

The authors declare that they have no known competing financial interests or personal relationships that could have appeared to influence the work reported in this paper.
